# Barrel-shaped design of the forearm free flap for lower lip reconstruction: a pilot case-control study

**DOI:** 10.1186/s12893-020-00792-x

**Published:** 2020-06-12

**Authors:** Nan-Chin Lin, Shih-Lung Lin, Kuo-Yang Tsai

**Affiliations:** 1 School of Dentistry, China Medical University, Taichung, Taiwan; 2 Department of Oral and Maxillofacial surgery, Changhua Christian Hospital, No. 235, Xuguang Rd., Changhua City, Changhua County 500 Taiwan; 3 Department of Plastic and Reconstructive Surgery, Changhua Christian Hospital, Changhua, Taiwan; 4 College of Nursing and Health Science, Da-Yeh University, Changhua, Taiwan

**Keywords:** Lower lip reconstruction, Forearm free flap, Quality of life, Lip defect surgery, Lower lip carcinoma surgery

## Abstract

**Background:**

Radial free forearm flaps is indicated patients with total or near-total defects in their lower lip. The purpose of our study was to evaluate a simple and effective barrel-shaped design of the radial free forearm flap for lower lip reconstruction and to compare its clinical outcomes with those of a conventional rectangular shaped free forearm flap.

**Methods:**

Twenty-two patients with a lower lip carcinoma who underwent radial forearm free flap reconstructive surgery were enrolled in this study between January 1, 2012, and December 31, 2017. A barrel-shaped design of radial forearm free flap for reconstruction was used in 8 patients (case group), and a rectangular design was used in 14 patients (control group). The patients’ quality of life was evaluated preoperatively and postoperatively in all the patients using the European Organization for Research and Treatment of Cancer Quality of Life (EORTC-HN35) questionnaire. We analyzed the differences in the EORTC QLQ-HN35 scores pre- and postoperatively between the case and control group.

**Results:**

The patients in the case group had better outcomes in swallowing, speech, social eating, social contact, and dry mouth than the control group at 1-year follow-up (*P* < 0.05).

**Conclusions:**

The use of a barrel-shaped design free forearm flap for lower lip reconstruction is an effective procedure and can achieve better results than the use of rectangular free forearm flap.

## Background

Complex lower lip defects can pose formidable challenges during reconstructive surgery after tumor excision, burn injury debridement, or facial trauma. Moreover, speech problems, oral incontinence, and poor cosmetic outcomes usually result from the loss of sensation in the lower lip and continuity with the orbicularis muscle of the lower lip. Small defects in the lower lip can be reconstructed using local flaps to obtain favorable functional and cosmetic outcomes. Several reconstruction methods have been reported for cases in which a defect in the lower lip exceeds two-thirds of the total lip volume, however, to the best of our knowledge, there is no simple procedure to achieve both competence of the oral cavity as well as aesthetic outcomes [1].

Full-thickness defects exceeding half of the lower lip volume cannot be closed with the remaining lip tissue without causing microstomia; however, reconstruction with local tissue can sometimes achieve good lower lip continuity. In cases of total or near-total defects in the lower lip, Sakai et al. (1989) have indicated the use of radial forearm free flaps in reconstructive surgery. The use of free flaps can provide distant and healthy tissues that are taken away from head and neck region; however, frequent drooling occurs owing to the lack of sensation in the flap and discontinuity of the orbicularis muscle [2].

To reconstruct a dynamic sphincter, several methods of suspension procedures using the tendo-fasciocutaneous flap have been described in the literature [3]. The method first described by Sakai et al. [2] has been used and modified by other researchers [4–6].. However, this type of reconstructive procedure has disadvantages, including sagging in the middle portion of the flap, particularly when the patients are eating or drinking which in turn causes drooling [4, 5]. Studies have also reported that approximately 15% of patients have no palmaris longus which is the most used muscle for suspension procedures [7, 8].

Drooling is an unacceptable social problem for patients. Thus, the purpose of our study was to evaluate a simple (without suspension) and effective (different shape) design of a free forearm flap for lower lip reconstruction and to further compare it with a conventional rectangular free forearm flap.

## Methods

### Patients

This case-control study was approved by the Institutional Review Board and Ethical Committee of Changhua Christian Hospital. All participants provided written informed consent regarding the publication of their images. The study identified 25 patients with lower lip carcinoma who underwent radial forearm free flap reconstructive surgery between January 1, 2012, and December 31, 2017. Patients who were lost to follow-up (*n* = 2) and who had a tumor in the corner of the mouth (*n* = 1) were excluded. Finally, 22 patients were included in the analysis. The barrel-shaped design of the radial forearm free flap for reconstruction was used in 8 patients (case group) and the rectangular design was used in the remaining 14 patients (control group).

### Evaluation of quality of life

The patients’ quality of life was evaluated preoperatively (t1 in the case group and t1’ in the control group) and postoperatively (1-year follow-up, t2 in the case group and t2’ in the control group) using the European Organization for Research and Treatment of Cancer Quality of Life questionnaire (EORTC QLQ-HN35) [9]. The detailed EORTC QLQ-HN35 and manual book are shown in Supplementary File 1 and Supplementary File 2. The EORTC QLQ-HN35 questionnaire is a 35-item module comprising 7 symptom scales (pain, swallowing, taste or smell, speech, social eating, social contacts, and sexuality) and 6 symptom items (teeth problems, trismus, dry mouth, sticky saliva, cough, and feeling ill) [10, 11]. The patients were instructed to rate the presence of a symptom or limitation of a function using the Likert-like four-point scale (not at all, a little, quite a bit, or very much). Five questions from the EORTC QLQ-HN35 questionnaire were answered with a yes or no [12]. The internal consistency of the included domains was adequate [13]. In addition, the EORTC QLQ-HN35 questionnaire is less susceptible to changes in the clinical status of the patients compared with other measurement tools [13]. In this study, the scores were transformed into 0–100 scale scores with a high score indicating a high level of symptoms or dysfunctions. Both these instruments have formally validated Taiwanese translations (Supplementary File 3) [14]. We analyzed the change in the EORTC QLQ-HN35 scores (t2-t1 score and t2’-t1’ score) between the case and control groups.

### Barrel-shaped design of the radial forearm free flap

**Fig. 1:**
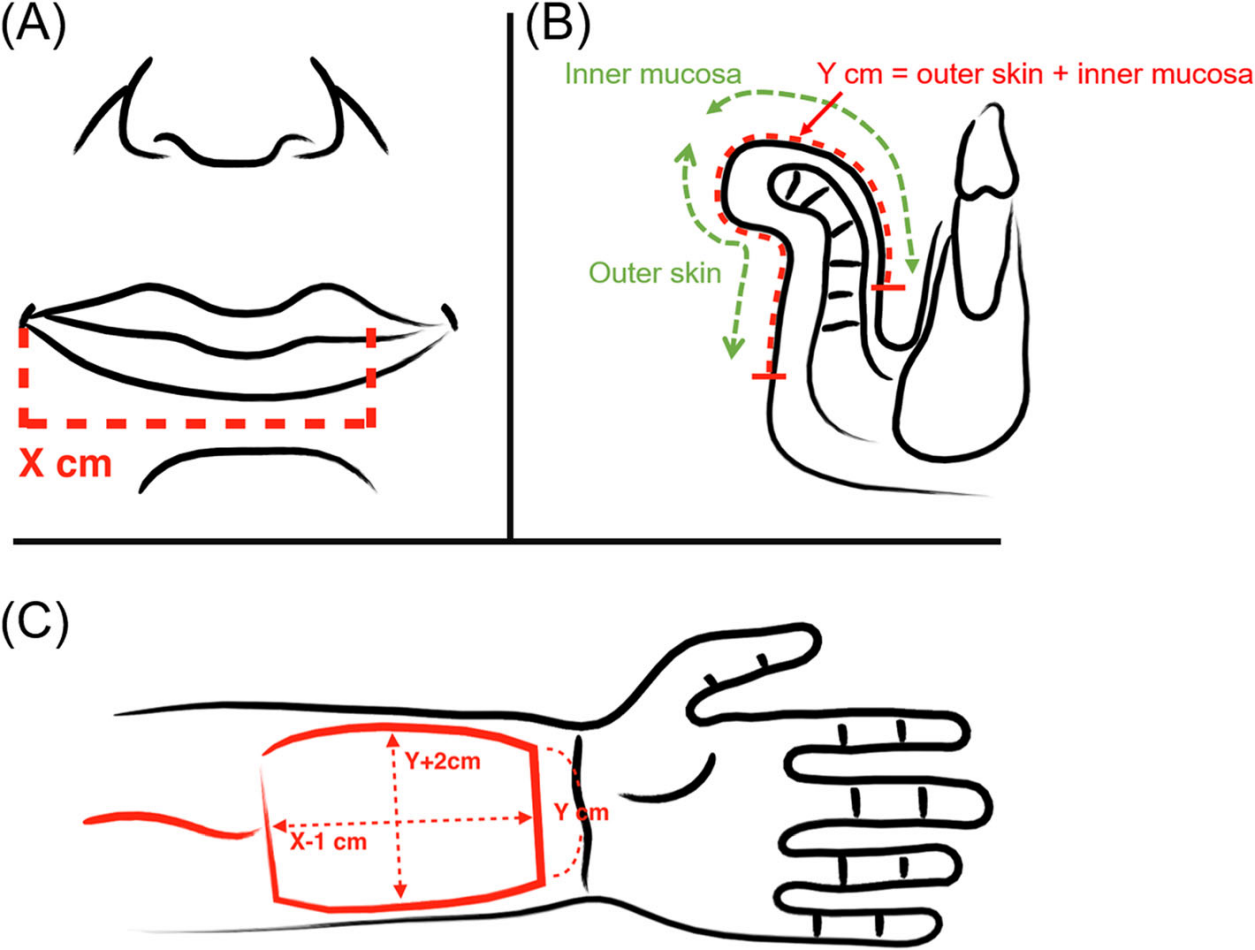
Illustrates the barrel-shaped design of the radial forearm free flap. (**a**) If the horizontal defect was “X” cm and (**b**) the vertical defect from the outer skin to the inner mucosa surface was “Y” cm. (**c**) The flap was harvested with a longitudinal dimension of X − 1 cm and with a transverse dimension of Y + 2 cm in a barrel-like shape

The present study introduces an effective design for large lower lip defect reconstruction using a free forearm flap without a tendon sling. First, the flap was designed in a barrel-like shape and folded along the direction of the pedicle to reconstruct both the mucosal lining and external skin (Fig. 1). This design resulted in a convex shape in the middle portion of the flap after folding. Second, if the horizontal defect was X cm, we harvested the flap with a longitudinal dimension of X − 1 cm (Fig. 1a and c). If the vertical defect, including the outer skin and inner mucosa, was Y cm, we harvested the flap with a transverse dimension of Y + 2 cm (Fig. 1b and c). Shortening the horizontal length can maintain some tension and prevent forward sagging of the flap. The vertical height of the flap must be lengthened because firstly, when the flap is plicated, its thickness will consume some skin length and secondly, to make the center of the flap higher, and similar to a normal lip and to prevent sagging of the middle portion of the flap.

In the control group, a rectangular shaped flap was harvested and the size was matched with that of the defect. For example, if the horizontal defect was X cm and the vertical defect, including the outer skin and inner mucosa, was Y cm, we harvested an X × Y cm flap.

### Statistical analysis

Continuous variables are presented as mean ± standard deviation and categorical variables as percentages. The Mann–Whitney’s U-test was used to compare continuous variables between the case and control groups. A *P*-value of < 0.05 was considered statistically significant. All statistical analyses were performed using the Statistical Package for the Social Sciences software for Windows (version 16, SPSS, Chicago, Il, USA).

## Results

**Fig. 2:**
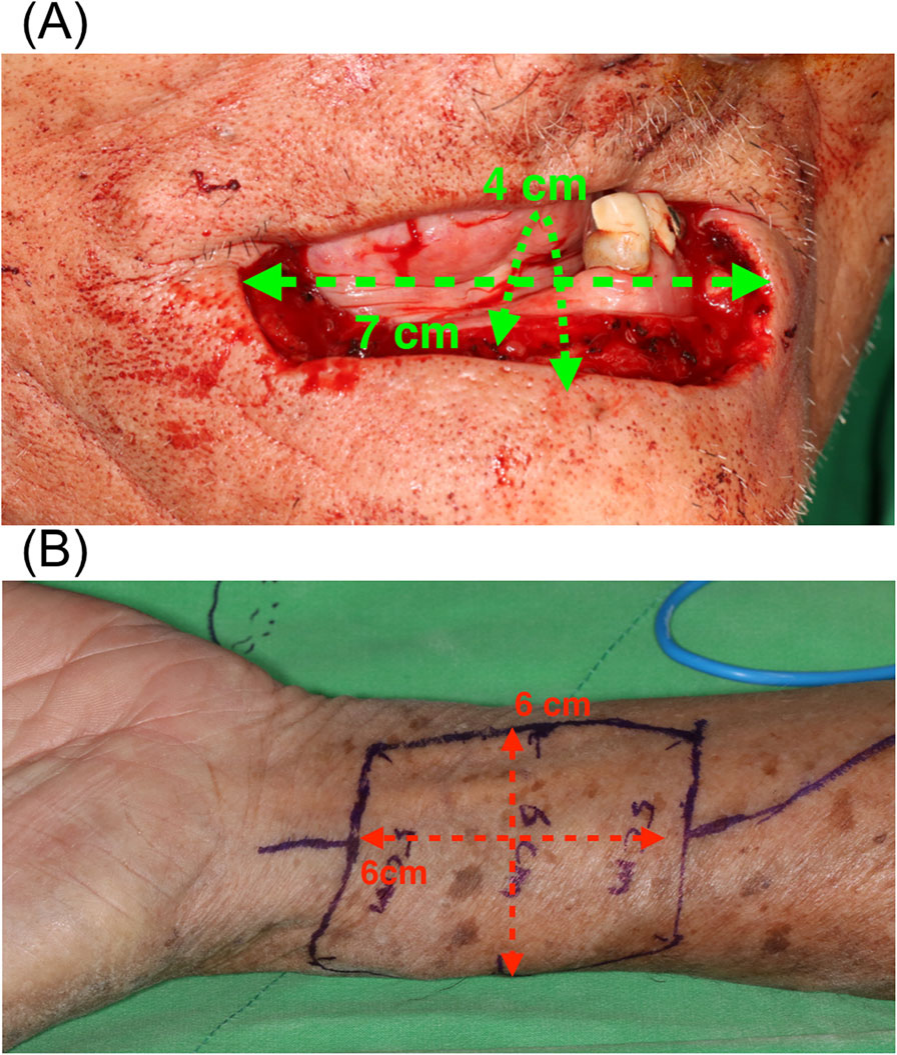
In the clinical case, **a** the horizontal defect was 7 cm and the vertical defect from the outer skin to the inner mucosa surface was 4 cm. **b** The free forearm flap was harvested in a barrel-like shape with a dimension of 6 × 6 cm

**Fig. 3:**
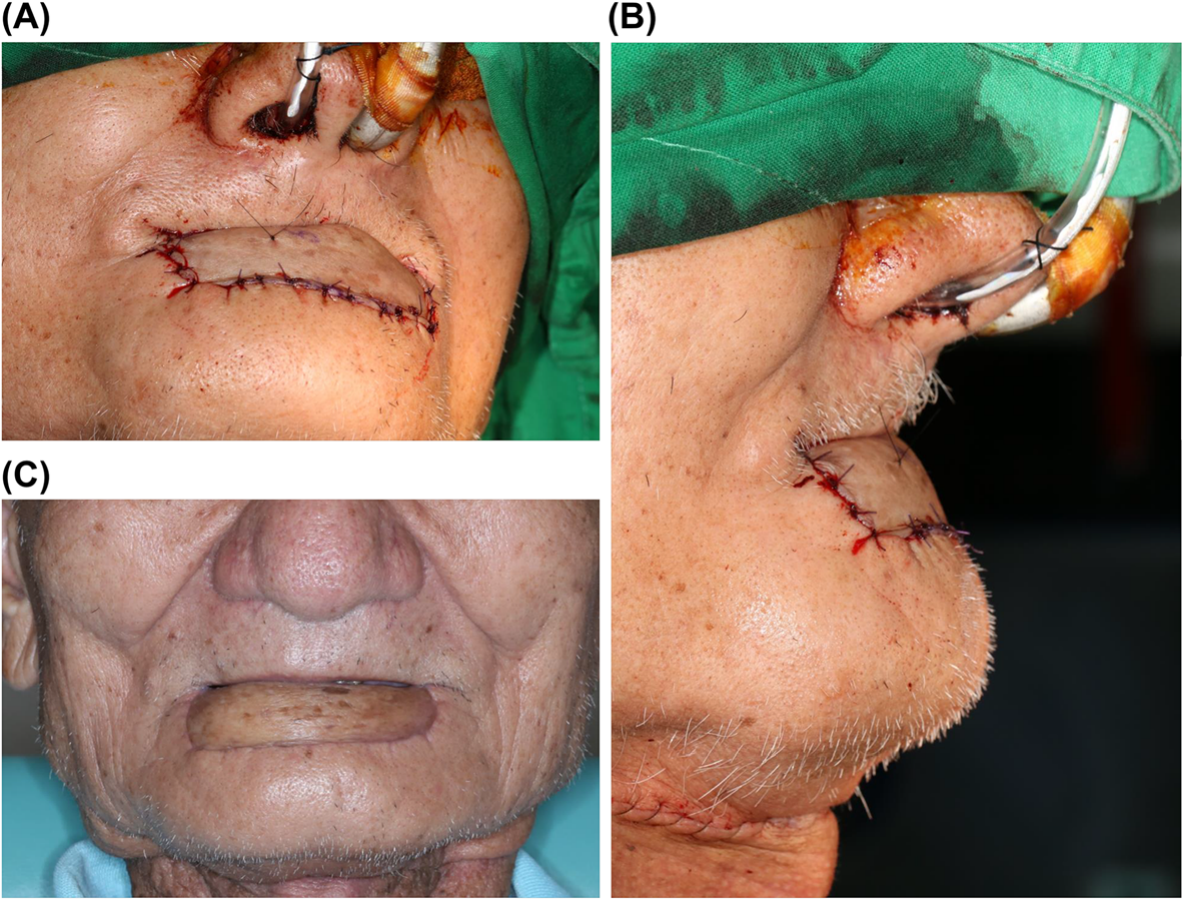
In the postoperative frontal view (**a**) and lateral view (**b**), the middle portion of the flap will be slightly convex to restore the oral competence. At the same time, shortening the longitudinal length of the harvested flap can maintain some tension to prevent sagging of the flap. (**c**) The image at the 1-year follow-up still shows a very good result in the frontal view

**Fig. 4:**
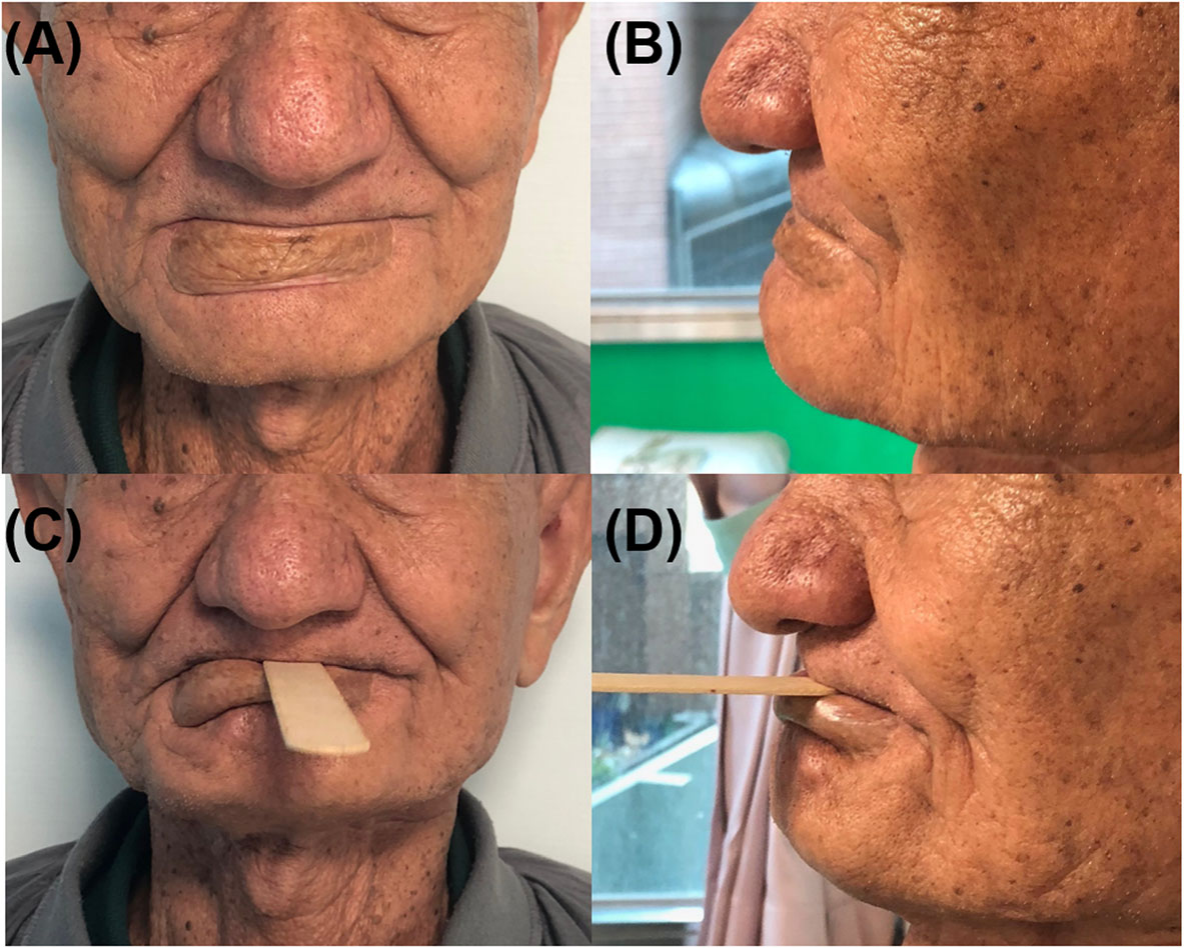
The image at the 2-year follow-up, the frontal view (**a**) and lateral view (**b**) show good oral competence after surgery. In the lip functional test, (**c**) and (**d**) show that the patient can use his lip to hold the stick showing that the barrel-shaped design of radial forearm free flap can restore oral competence

In our clinical case, the horizontal defect was 7 cm and the vertical defect was 4 cm (including the outer skin and inner mucosa), respectively (Fig. 2a). Therefore, the free forearm flap was designed in a barrel-like shape with dimensions of 6 × 6 cm (Fig. 2b). The postoperative and 1-year follow-up images of the patient are shown in Fig. 3. The patient was eating a normal diet and had good oral competence by the second-year of follow-up (Fig. 4).

The patient characteristics of both groups are summarized in Table 1. The characteristics, including age, sex, body mass index, postoperative radiotherapy, tumor staging, defect size, alcohol consumption, smoking, and history of betel nut chewing, did not differ between the two groups. All the flaps were harvested and transferred without any complications. No case of donor site infection or wound dehiscence was observed.

**Table 1:** Clinical characteristics of both groups

	Case ***n*** = 8 (%)	Control ***n*** = 14 (%)
**Age (years), mean**	61.25 ± 8.44	57.86 ± 6.03
**Sex: Male**	8 (100.0)	10 (90.9)
**Female**	0 (0)	1 (9.1)
**Body mass index**	22.84 ± 3.52	24.57 ± 4.73
**Postoperative radiotherapy**	0 (0)	1 (9.1)
**T status: T1**	4 (50.0)	10 (71.4)
**T2**	4 (50.0)	2 (14.3)
**T3**	0 (0)	2 (14.3)
**N status: N0**	8 (100.0)	14 (100.0)
**M status: M0**	8 (100.0)	14 (100.0)
**Defect size: More than or equal to 7 × 7 cm**	1 (12.5)	3 (21.4)
**6 × 6 cm to 7 × 7 cm**	5 (62.5)	7 (50.0)
**Smaller than 6 × 6 cm**	2 (25.0)	4 (28.6)
**Alcohol consumption: Yes**	4 (50.0)	10 (71.4)
**No**	4 (50.0)	4 (28.6)
**Smoking: Yes**	8 (100.0)	12 (85.7)
**No**	0 (0)	2 (14.3)
**Betel nut chewing: Yes**	8 (100.0)	14 (100.0)
**No**	0 (0)	0 (0)

The patients were evaluated preoperatively and postoperatively at the 1-year follow-up. Table 2 summarizes the results of the analyses of both the groups. Patients in the case group had better outcomes in swallowing, speech, social eating, social contact, and dry mouth than the control group (*P* < 0.05), whereas no significant differences were identified for pain, sense, sexuality, teeth, open mouth, sticky saliva, cough, pain killer, nutritional supplements, feeding tube, and gain or loss of weight.

**Table 2:** EORTC QOL-HN 35 scores were evaluated preoperatively (t1 in case group and t1’ in control group) and post-operatively (1-year follow up, t2 in case group and t2’ in control group). Mean (standard deviation), Median (interquartile range) and *p* values (Mann-Whitney U test) of the change in EORTC QOL-HN 35 scores over time between t1 and t2, and between t1’ and t2’

Changes in EORTC QOL-HN 35 score	EORTC QOL-HN 35 scores								***p***-value
	Case, n = 8				Control, n = 14				
	t1 (Mean)	t2 (Mean)	t2 − t1 (Mean)	t2 − t1 (Median)	t1’ (Mean)	t2’ (Mean)	t2’ − t1’ (Mean)	t2’ − t1’ (Median)	
**Pain**	31.250	34.375	3.125 ± 5.786	0 (0–6.250)	33.750	32.857	−0.893 ± 3.144^a^	0 (−6.250–0)	0.1251
**Swallowing**	25.0	28.125	3.125 ± 3.341	3.125 (0–6.250)	31.25	60.714	29.464 ± 14.998	25.0 (18.750–43.750)	**0.0001**
**Sense**	25.0	28.125	3.125 ± 5.786	0 (0–6.250)	25.0	35.714	10.714 ± 22.391	0 (0–12.50)	0.7409
**Speech**	25.0	29.167	4.167 ± 4.454	4.167 (0–8.333)	26.189	52.379	26.190 ± 24.646	25.0 (8.333–4.167)	**0.0119**
**Social eating**	28.125	43.750	15.625 ± 13.774	9.375 (6.25–25.0)	30.357	60.714	30.357 ± 14.052	25.0 (18.750–50.0)	**0.0197**
**Social contact**	25.0	43.750	18.75 ± 4.432	17.50 (15.0–22.5)	25.0	52.571	27.571 ± 9.982	25.0 (20.0–35.0)	**0.039**
**Sexuality**	25.0	31.250	6.250 ± 11.573	0 (0–12.5)	25.0	35.714	10.714 ± 18.898	0 (0–25.0)	0.7409
**Teeth**	31.250	37.50	6.250 ± 11.573	0 (0–12.5)	39.286	46.429	7.143 ± 11.720	0 (0–25.0)	0.8678
**Open mouth**	37.50	37.50	0	0	39.286	50.0	10.714 ± 18.898	0 (0–25.0)	0.1370
**Dry mouth**	31.250	43.750	12.50 ± 13.363	12.50 (0–25.0)	28.571	57.142	28.571 ± 9.078	25.0 (25.0–25.0)	**0.0082**
**Sticky saliva**	25.0	25.750	0.750 ± 0.886	0.50 (0–1.50)	25.0	39.286	14.286 ± 12.839	25.0 (0–25.0)	0.1503
**Cough**	25.0	25.0	0	0	25.0	28.571	3.571 ± 9.078	0	0.3603
**Questionnaire 31–35** **summed up**	50.0	52.5	2.50 ± 3.273	1.25 (0–5.0)	51.429	51.429	0	0	0.1094

*p*-value by Mann–Whitney’s U-test

^a^ A negative value indicates pain relief

## Discussion

The importance of the lips as an anatomical structures goes beyond its vital functions and includes eating, drinking, phonation, speaking, and expressing emotions. In cases of lip carcinoma, reconstruction of the lower lip often requires a complex surgical approach to best achieve the goal of restoring oral competence and maintaining an adequate oral aperture to facilitate oral hygiene [15]. In the present study, the barrel-shaped design used to reconstruct the lower lip defect achieved better outcomes in swallowing, speech, social eating, social contact, and dry mouth than the rectangular free forearm flap. The barrel-shaped free forearm flap can achieve good oral competence and enable patients to return to a normal diet with one-stage reconstructive surgery without any additional suspension procedures.

Drooling is an unacceptable problem that not only affects eating, drinking, and swallowing but also results in social problems [5]. It is well-known that achieving oral competence is the key to the prevention of drooling. In the present study, we also evaluated drooling using the EORTC QLQ-HN35 scores in the “Swallowing” and “Social eating” sections. In the “Swallowing” section, there were three of four questions about drooling including “Have you had a problem eating liquids, pureed food, or solid food?” In the “Social eating” section, there were four questions for patients about drooling including “Have you had trouble eating or enjoying meals?” and “Have you had trouble eating in front of your family or in front of other people?” In the case group, the increased scores in the “Swallowing” and “Social eating” sections were significantly lower than those of the control group. We can reasonably presume that the oral competence of in the case group was better than that of the control group. This key point resulted in better functions of swallowing, speaking, and social contact (Table 2). However, when intra-group comparisons were performed, we observed that the EORTC QLQ-HN35 scores for both control and case groups were getting worse in function and social problem pre-operation to post-operation. However, more cases and data must be collected to approve this finding in the future. To reconstruct a dynamic sphincter, several suspension procedures using the free forearm flap have been reported in the literature [2–6]. Sakai et al. [2] described the use of the palmaris longus tendon with a free forearm flap for full-thickness defects of the lower lip. The tendon was fixed to the orbicularis oris muscle and epidermis of the nasolabial area; however, the suspension procedure could not maintain oral competence while the patients were eating or speaking [5]. Jeng et al. modified the method described by Sakai et al. [5]. They harvested the free forearm flap according to the defect size without any modification as well as harvested the palmaris longus tendon which is sutured to the remaining orbicularis muscle and the angles of the mouth. However, the use of the suspension procedure for reconstruction has well-known disadvantages. First, approximately 15% of patients have no palmaris longus which is the most commonly used muscle for suspension procedures [7, 8]. Second, the procedure has a longer operation time. Finally, the middle portion of the suspended flap sags. In comparison, the barrel-shaped design of the free forearm flap, when folded, is slightly convex in the middle portion of the flap. This can help in achieving oral competence, as shown in our clinical case. The use of a barrel-shaped free forearm flap does not result in a longer operating time.

The present study has several limitations. First, the small sample size might have prevented the identification of significant differences between the case and control groups. Second, its retrospective design might have increased the risk of bias in case selection. Finally, we did not compare the outcomes between the barrel-shaped design of the free forearm flap and a procedure involving suspension. Therefore, further studies are warranted to determine this.

## Conclusion

In conclusion, we demonstrate that a barrel-shaped design free forearm flap for lower lip reconstruction achieves better outcomes than a rectangular design for function and social contact evaluated by an EORTC QLQ-HN35 questionnaire. We found that the use of this barrel-shaped forearm free flap for lower lip reconstruction is an effective procedure, which can improve oral competence and prevent drooling.

## Supplementary information

**Additional file 1 : Supplementary File 1.** European Organization for Research and Treatment of Cancer Quality of Life questionnaire (EORTC QLQ-HN35) in English version.

**Additional file 2 : Supplementary File 2.** Manual book of EORTC QLQ-HN35 questionnaire.

**Additional file 3 : Supplementary File 3.** EORTC QLQ-HN35 questionnaire in Taiwanese version.

## Data Availability

The datasets used and analyzed during the current study are available from the corresponding author on reasonable request.

## Abbreviations

EORTC QLQ-HN35: European Organization of Research and Treatment of Cancer Quality of Life head and neck 35 questionnaire.
