# Supplementary material for: Barrel-shaped design of the forearm free flap for lower lip reconstruction: a pilot case-control study
Source: BMC Surg. 2020 Jun 12;20:132. doi: 10.1186/s12893-020-00792-x (PMC7291574; doi:10.1186/s12893-020-00792-x)
Supplement: Supplementary file 1 — Additional file 1 : Supplementary File 1. European Organization for Research and Treatment of Cancer Quality of Life questionnaire (EORTC QLQ-HN35) in English version. [file 12893_2020_792_MOESM1_ESM.pdf]

EORTC QLQ - H&N35

Patients sometimes report that they have the following symptoms or problems. Please indicate the extent to which you have experienced these symptoms or problems during the past week. Please answer by circling the number that best applies to you.

**During the past week:**	**Not at all**	**A little**	**Quite a bit**	**Very much**
31. Have you had pain in your mouth?	1	2	3	4
32. Have you had pain in your jaw?	1	2	3	4
33. Have you had soreness in your mouth?	1	2	3	4
34. Have you had a painful throat?	1	2	3	4
35. Have you had problems swallowing liquids?	1	2	3	4
36. Have you had problems swallowing pureed food?	1	2	3	4
37. Have you had problems swallowing solid food?	1	2	3	4
38. Have you choked when swallowing?	1	2	3	4
39. Have you had problems with your teeth?	1	2	3	4
40. Have you had problems opening your mouth wide?	1	2	3	4
41. Have you had a dry mouth?	1	2	3	4
42. Have you had sticky saliva?	1	2	3	4
43. Have you had problems with your sense of smell?	1	2	3	4
44. Have you had problems with your sense of taste?	1	2	3	4
45. Have you coughed?	1	2	3	4
46. Have you been hoarse?	1	2	3	4
47. Have you felt ill?	1	2	3	4
48. Has your appearance bothered you?	1	2	3	4

SPECIMEN

Please go on to the next page

During the past week:	Not at all	A little	Quite a bit	Very much
49. Have you had trouble eating?	1	2	3	4
50. Have you had trouble eating in front of your family?	1	2	3	4
51. Have you had trouble eating in front of other people?	1	2	3	4
52. Have you had trouble enjoying your meals?	1	2	3	4
53. Have you had trouble talking to other people?	1	2	3	4
54. Have you had trouble talking on the telephone?	1	2	3	4
55. Have you had trouble having social contact with your family?	1	2	3	4
56. Have you had trouble having social contact with friends?	1	2	3	4
57. Have you had trouble going out in public?	1	2	3	4
58. Have you had trouble having physical contact with family or friends?	1	2	3	4
59. Have you felt less interest in sex?	1	2	3	4
60. Have you felt less sexual enjoyment?	1	2	3	4

During the past week:	No	Yes
61. Have you used pain-killers?	1	2
62. Have you taken any nutritional supplements (excluding vitamins)?	1	2
63. Have you used a feeding tube?	1	2
64. Have you lost weight?	1	2
65. Have you gained weight?	1	2

SPECIMEN

© Copyright QLQ-H&N35 1994 EORTC Quality of Life Group, version 1. All rights reserved
