# Supplementary material for: Barrel-shaped design of the forearm free flap for lower lip reconstruction: a pilot case-control study
Source: BMC Surg. 2020 Jun 12;20:132. doi: 10.1186/s12893-020-00792-x (PMC7291574; doi:10.1186/s12893-020-00792-x)
Supplement: Supplementary file 2 — Additional file 2 : Supplementary File 2. Manual book of EORTC QLQ-HN35 questionnaire. [file 12893_2020_792_MOESM2_ESM.pdf]

Head & Neck cancer module: QLQ-H&N35

The head & neck cancer module is meant for use among a wide range of patients with head & neck cancer, varying in disease stage and treatment modality (i.e. surgery, radiotherapy and chemotherapy) (Bjordal and Kaasa, 1992; Bjordal *et al.*, 1994, 1999, 2000). The module comprises 35 questions assessing symptoms and side effects of treatment, social function and body image/sexuality (Appendix 2b). The module has been developed according to the guidelines, and pretested on patients from Norway, Sweden, Denmark, the UK and French-speaking Belgium. It has been field tested in Norway, Sweden and The Netherlands, and in a large cross-cultural study involving more than ten countries (EORTC Protocol 15941).

Scoring of the head & neck cancer module

The head & neck cancer module incorporates seven multi-item scales that assess pain, swallowing, senses (taste and smell), speech, social eating, social contact and sexuality. There are also eleven single items. For all items and scales, high scores indicate more problems (i.e. there are no function scales in which high scores would mean better functioning).

The scoring approach for the QLQ-H&N35 is identical in principle to that for the symptom scales / single items of the QLQ-C30.

Scale name	Scale	Number of items	Item range*	QLQ-H&N35 Item numbers
**Symptom scales / items**				
Pain	HNPA	4	3	1 – 4
Swallowing	HNSW	4	3	5 – 8
Senses problems	HNSE	2	3	13,14
Speech problems	HNSP	3	3	16,23,24
Trouble with social eating	HNSO	4	3	19 – 22
Trouble with social contact	HNSC	5	3	18,25 – 28
Less sexuality	HNSX	2	3	29,30
Teeth	HNTE	1	3	9
Opening mouth	HNOM	1	3	10
Dry mouth	HNDR	1	3	11
Sticky saliva	HNSS	1	3	12
Coughing	HNCO	1	3	15
Felt ill	HNFI	1	3	17
Pain killers	HNPK	1	1	31
Nutritional supplements	HNNU	1	1	32
Feeding tube	HNFE	1	1	33
Weight loss	HNWL	1	1	34
Weight gain	HNWG	1	1	35

* "Item range" is the difference between the possible maximum and the minimum response to individual items.
