# Supplementary material for: Barrel-shaped design of the forearm free flap for lower lip reconstruction: a pilot case-control study
Source: BMC Surg. 2020 Jun 12;20:132. doi: 10.1186/s12893-020-00792-x (PMC7291574; doi:10.1186/s12893-020-00792-x)
Supplement: Supplementary file 3 — Additional file 3 : Supplementary File 3. EORTC QLQ-HN35 questionnaire in Taiwanese version. [file 12893_2020_792_MOESM3_ESM.pdf]

附件五 生活品質量表

說明：此次住院階段裡，您多常有以下問題?請將最接近您的感受打『v』

項目		很常有(4)	偶爾有(3)	不常有(2)	從來沒有(1)
**疼痛問題**					
1	口腔疼痛				
2	下顎疼痛				
3	口腔腔酸痛				
4	喉嚨痛				
**吞嚥問題**					
5	吞嚥液體時曾有困難				
6	吞嚥濃湯時曾有困難				
7	吞嚥固體食物時曾有困難				
**8**	吞嚥時曾經嗆到				
**感覺問題**					
9	曾有嗅覺方面的問題				
10	曾有味覺方面的問題				
**語言問題**					
11	曾有聲音沙啞				
12	與別人交談感到困擾				
13	用電話交談接觸曾感到困擾				
**社交飲食問題**					
14	進食困擾				
15	在家人面前進食困擾				
16	在其他人面前進食困擾				
17	難以享受進餐的樂趣				
**社會接觸問題**					
18	外觀困擾				
19	和家人交往接觸曾感到困擾				
20	和朋友交往接觸曾感到困擾				
21	外出到公共場合曾感到困擾				
22	和家人或朋友有身體接觸時，曾感到困擾				
**性生活問題**					
23	對「性」有沒有樂趣				

項　　　　　　　　目		很常有(4)	偶爾有(3)	不常有(2)	從來沒有(1)
24	感到比較無法享受性生活				

說明：此次住院階段裡，您是否有以下問題?或是否接受止痛藥或營養劑或餵食管，請將您的狀況打『v』

項　　　　　　　　目		有（2）	沒有（1）
25	牙齒方面的問題		
26	張口問題		
27	口乾問題		
28	唾液黏稠問題		
29	咳嗽問題		
30	病態感		
31	使用止痛藥		
32	使用營養劑		
33	使用餵食管		
34	體重減輕		
35	體重增加		
